# Prevalence and risk factors of having antibodies to class I and II human leukocyte antigens in older haploidentical allograft candidates

**DOI:** 10.1038/s41598-020-59417-1

**Published:** 2020-02-11

**Authors:** Le-Qing Cao, Meng Lv, Lan-Ping Xu, Xiao-Hui Zhang, Huan Chen, Yu-Hong Chen, Feng-Rong Wang, Wei Han, Yu-Qian Sun, Chen-Hua Yan, Fei-Fei Tang, Xiao-Dong Mo, Kai-Yan Liu, Xiao-Jun Huang, Ying-Jun Chang

**Affiliations:** 1 Peking University People’s Hospital and Peking University Institute of Hematology, Beijing Key Laboratory of Hematopoietic Stem Cell Transplantation, No. 11 South Street of Xizhimen, Xicheng District, Beijing, 100044 P.R. China; 2 Peking-Tsinghua Center for Life Sciences, Beijing, 100871 China; 3 Collabrative Innovation Center of Hematology, Peking University, Beijing, 100044 China

**Keywords:** Autoimmunity, Bone marrow transplantation, Haematological diseases

## Abstract

The effect of donor-specific anti-human leukocyte antigen (HLA) antibodies (DSAs) has been recognized as a factor in graft failure (GF) in patients who underwent umbilical cord blood transplantation (UBT), matched unrelated donor transplantation (MUDT), or haploidentical stem cell transplantation (haplo-SCT). Presently, we know little about the prevalence of and risk factors for having anti-HLA antibodies among older transplant candidates. Therefore, we analyzed 273 older patients with hematologic disease who were waiting for haplo-SCT. Among all patients, 73 (26.7%) patients had a positive panel-reactive antibody (PRA) result for class I, 38 (13.9%) for class II, and 32 (11.7%) for both. Multivariate analysis showed that females were at a higher risk for having a PRA result for class II (*P* = 0.001) and for having antibodies against HLA-C and HLA-DQ. Prior pregnancy was a risk factor for having a PRA result for class I (*P* < 0.001) and for having antibodies against HLA-A, HLA-B and HLA-DQ. Platelet transfusions were risk factors for the following: having a positive PRA result for class I (*P* = 0.014) and class II (*P* < 0.001); having antibodies against HLA-A, HLA-B, HLA-C, HLA-DP, HLA-DQ, and HLA-DR; and having higher mean fluorescence intensity (MFI) of PRA for class I (*P* = 0.042). In addition, previous total transfusions were at high risk for having higher numbers of antibodies to specific HLA loci (*P* = 0.005), and disease course (7.5 months or more) (*P* = 0.020) were related to higher MFI of PRAs for class I. Our findings indicated that female sex, prior pregnancy, platelet transfusions and disease courses are independent risk factors for older patients with hematologic disease for having anti-HLA antibodies, which could guide anti-HLA antibody monitoring and be helpful for donor selection.

## Introduction

Allogeneic hematopoietic stem cell transplantation (allo-HSCT) is recognized as an effective therapy for the majority of malignant hematologic diseases^1–4^. However, primary and secondary graft failure (GF) remain a serious complication of allo-HSCT. Multiple factors have been implicated in GF, such as the primary diagnosis, advanced disease status, conditioning regimens, and stem cell dose^5–8^. In recent years, the effects of donor-specific anti-human leukocyte antigen (HLA) antibodies (DSAs) have been recognized as a factor in GF^9–11^, either in patients who underwent umbilical cord blood transplantation (UBT), matched unrelated donor transplantation (MUDT), or haploidentical stem cell transplantation (haplo-SCT)^10–16^. Therefore, DSAs can influence who is the best donor in HLA-mismatched allo-HSCT settings^17,18^.

Given the importance of anti-HLA antibodies, there have been many studies focusing on the prevalence and risk factors that may lead to the development of anti-HLA antibodies. Hung *et al*.^19^ found that pregnancy and recent transfusion are independent risk factors for HLA sensitization in patients with end-stage renal disease. Akgul *et al*.^20^ showed that in patients who are waiting for kidney transplantation, sensitization by pregnancy and transplantation have a significant impact on the development of HLA class I and class II antibodies. A previous study by our group showed that the risk factors associated with the prevalence of panel-reactive antibody (PRA), either for class I or class II HLA, in transplant candidates were female sex, prior transfusions, pregnancy and myelodysplastic syndrome (MDS)^21^. In pediatric transplant recipients, we also confirmed that a diagnosis of MDS was an independent risk factor for a higher incidence of PRAs for both class I and II^22^. However, there have been few studies on the prevalence and risk factors for PRA among older patients. Therefore, we prospectively analyzed 297 older patients with hematological diseases who were waiting for HSCT to investigate the prevalence and risk factors for having anti-HLA antibodies.

## Materials and Methods

### Ethics statement

This study met the guidelines of the Helsinki Declaration, and was approved by the ethics committee of Ethic Committee of Peking University People’s Hospital (2015PHB010-01). Informed consent was obtained from all patients or their guardians and donors. This study was registered at http://www.chictr.org.cn/ ChiCTR-OPC-15006672.

### Patients

Between January 2015 and August 2018, 273 older candidates (aged more than 50 years) with hematological diseases who were waiting for HSCT were prospectively enrolled in this study (Table 1). The test results for anti-HLA antibody, the potential risk factors that may cause the development of anti-HLA antibodies, such as prior transfusion, pregnancy and disease courses, were collected.

**Table 1:** Demographic and Clinical Characteristics.

All patients	273
Median age(range), years	54(50–66)
Gender, n (%)	
Male	165(60.4%)
Female	108(39.6%)
Diagnosis, n (%)	
AML	124(45.4%)
ALL	47(17.2%)
MDS	66(24.2%)
CMML	10(3.7%)
Others	26(9.5%)
Number of pregnancies, n (%)	
0	168(61.5%)
1	61(22.3%)
≥2	44(16.1%)
Number of transfusions, n (%)	
≤12	212(77.7%)
>12	61(22.3%)
Number of RBC transfusion, n (%)	
≤7	212(77.7%)
>7	61(22.3%)
Number of platelet transfusion, n (%)	
≤7	226(82.8%)
>7	47(17.2%)
Course, n (%)	
≤7.5	169(61.9%)
>7.5	104(38.1%)
**PRA**	
Class I (+), n (%)	73(26.7%)
Class II (+), n (%)	38(13.9%)
Class I and II positive, n (%)	32(11.7%)
Class I or II positive, n (%)	79(28.9%)
Anti-HLA antibodies against single locus positive	79(28.9%)
Median MFI (range)	
Class I (+)	2902(558–22827)
Class II (+)	4332(516–18363)

Abbreviations: AML, acute myeloid leukemia; ALL, acute lymphoblastic leukemia; MDS, myelodysplastic syndrome; CMML, chronic myelomonocytic leukemia; RBC, red blood cell; PRA, panel reactive antibody; HLA, human leukocyte antigen; MFI, mean fluorescence intensity.

### Anti-HLA antibody detection

Patient serum was collected before transplantation and was screened for the presence of class I and class II anti-HLA antibodies (HLA abs) of the immunoglobulin G type with a LABScreen Mixed Kit (One Lambda, Canoga Park, CA, USA) according to the manufacturer’s instructions and as previously reported^9^. The mean fluorescence intensity (MFI) of the anti-HLA antibodies was adjusted for the background signal using the formula described previously. Samples with an MFI of 500 or more considered to be positive.

### Statistical analysis

Descriptive statistics, including the frequency (proportions) for categorical variables and median (range) or cut-off value determined by a ROC curve for quantitative variables, were used to describe the patient demographic and clinical characteristics. A chi-square test (and Fisher’s exact test, when appropriate) was used for group comparisons. Binary logistic regression tests with either univariate or multivariate analyses were performed to estimate the potential influence of the factors on anti-HLA antibodies. A two-sided P-value less than 0.05 was considered statistically significant. Statistical calculations were performed with SPSS, version 16.0 (Mathsoft, Seattle, WA, USA).

## Results

### Patient characteristics

The demographics and clinical characteristics are outlined in Table 1. In this study, 273 transplant candidates on waiting lists for haplo-SCT were analyzed for the presence of anti-HLA antibodies, including 165 males (60.4%) and 108 females (39.6%). There were 124 patients (45.4%) with acute myeloid leukemia (AML), 47 patients (17.2%) with acute lymphoblastic leukemia (ALL), 66 patients (24.2%) with myeloid dysplastic syndrome (MDS), 10 patients (3.7%) with chronic myelomonocytic leukemia (CMML) and 26 patients (9.5%) with other hematological diseases. The median age of the patients was 54 years (range, 50–66 years). The median MFI of panel-reactive antibodies (PRAs) for class I and class II were 2902 (range: 558–22827) and 4332 (range: 516–18363), respectively.

### Prevalence of PRAs for class I and/or class II

As shown in Table 1, 79 patients (28.9%) had a positive panel-reactive antibody (PRA) result for either class I or class II, 73 (26.7%) had a positive PRA result for class I, 38 (13.9%) had a positive PRA result for class II, and 32 (11.7%) had a positive PRA result for both class I and class II in this study.

### Risk factors associated with PRAs for class I and/or class II

Possible factors (including gender, age, diagnosis, number of pregnancies, number of total transfusions [including red blood cell (RBC) and platelet (PLT)], number of RBC transfusions, number of PLT transfusions and disease course) that may have influenced the PRAs were examined in univariate analysis (Supplementary Table S1). The risk factors associated with anti-HLA class I PRAs were sex, the number of pregnancies, number of total transfusions, number of RBC transfusions, number of PLT transfusions and disease course. Sex, the number of pregnancies, number of total transfusions, number of RBC transfusions and number of PLT transfusions were related to having anti-HLA class II PRAs. Sex, the number of pregnancies, number of total transfusions, number of RBC transfusions, number of PLT transfusions and disease course were related to having anti-HLA class I or II PRAs. Meanwhile, sex, the number of pregnancies, the number of total transfusions, number of RBC transfusions and number of PLT transfusions were associated with having anti-HLA class I and II PRAs. Multivariate analysis showed the following: (i) both prior pregnancy and PLT transfusions (7 or more) were associated with the prevalence of PRAs for class I (*P* < 0.001, *P* = 0.014) and for class I or II (*P* < 0.001, *P* = 0.006); (ii) both female sex and PLT transfusions (7 or more) were associated with a higher prevalence of PRAs for class II (*P* = 0.001, *P* < 0.001) and for class I and class II (*P* = 0.002, *P* < 0.001) (Table 2).

**Table 2:** Logistic regression analysis of PRA in 273 allogeneic stem cell transplantation candidates.

	Class I (+)			Class II (+)			Class I or II (+)			Class I and II (+)		
	HR	95% CI	P	HR	95% CI	P	HR	95% CI	P	HR	95% CI	P
Gender (male vs. female)			NS	3.719	1.751–7.900	0.001			NS	3.521	1.574–7.878	0.002
Number of pregnancies												
0	0.358	0.158–0.813	<0.001			NS	0.290	0.127–0.663	0.003			NS
1	0.191	0.093–0.393	<0.001				0.154	0.075–0.320	<0.001			
≥2		1			1			1			1	
Number of total transfusions(≤12 vs. >12)			NS			NS			NS			NS
Number of RBC transfusion(≤7 vs. >7)			NS			NS			NS			NS
Number of PLT transfusion(≤7 vs. >7)	2.365	1.188–4.710	0.014	5.070	2.321–11.074	<0.001	2.602	1.307–5.177	0.006	5.105	2.252–11.572	<0.001
Course(≤7.5 vs. >7.5)			NS			NS			NS			NS

Abbreviations: PRA, panel reactive antibody; OR, odds ratio; CI, confidence interval; RBC, red blood cell; PLT, platelet.

### Prevalence of antibodies against HLA-A, -B, -C, -DP, -DQ and -DR loci

**Figure 1:**
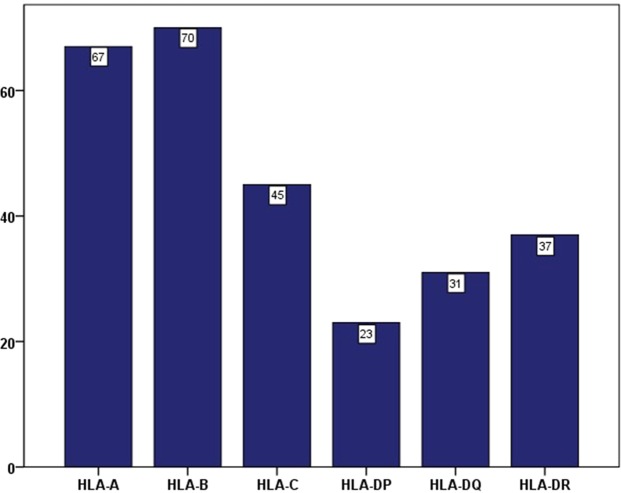
Frequency of anti-HLA antibodies against single locus in 273 older patients.

As shown in Fig. 1, among all 273 patients, the distribution of antibodies against different antigens demonstrated that 67 patients had antibodies against HLA-A (24.5%), 70 had antibodies against HLA-B (25.6%), 45 had antibodies against HLA-C (16.5%), 23 had antibodies against HLA-DP (8.4%), 31 had antibodies against HLA-DQ (11.4%) and 37 had antibodies against HLA-DR (13.6%).

### Risk factors associated with having antibodies against the HLA-A, -B, -C, -DP, -DQ and -DR loci

Univariate analysis showed that sex, prior pregnancy and PLT transfusions were risk factors for having anti-HLA antibodies against all loci. Previous total transfusions, and RBC transfusions were associated with having antibodies against HLA-A, B, -DP, -DQ and -DR. Meanwhile, the course of disease was related to having antibodies against HLA-B and -DP (Supplementary Table S2). Multivariate analysis showed that the following: i) female sex was associated with the development of antibodies against HLA-C (*P* = 0.003) and -DQ (*P* = 0.048); ii) prior pregnancy was independently and significantly related to the development of antibodies against HLA-A (*P* < 0.001), -B (*P* < 0.001) and -DQ (*P* = 0.001); iii) PLT transfusions (7 or more) were associated with having a higher prevalence of antibodies against HLA-A (*P* = 0.011), -B (*P* = 0.021), -C (P = 0.010), -DP (*P* < 0.001), -DQ (*P* < 0.001) and -DR (*P* < 0.001). In addition, it seemed that gender had a trend towards influencing the development of antibodies against HLA-DQ (*P* = 0.055) (Table 3).

**Table 3:** Specific antibodies against HLA-A, -B, -C, -DP, -DQ, and -DR: logistic regression analysis in 273 older allogeneic stem cell transplantation candidates.

	Anti-HLA-A		Anti-HLA-B		Anti-HLA-C		Anti-HLA-DP		Anti-HLA-DQ		Anti-HLA-DR	
	HR (95% CI)	P	HR (95% CI)	P	HR (95% CI)	P	HR (95% CI)	P	HR (95% CI)	P	HR (95% CI)	P
Gender (female vs. male)		NS		NS	2.729(1.404–5.303)	0.003		NS	12.615(1.020–156.051)	0.048		NS
**Number of pregnancies**												
0	0.402(0.174–0.25)	0.036	0.358(0.158–0.813)	0.014				NS	0.254(0.082–0.790)	0.018		NS
1	0.243(0.118–0.501)	<0.001	0.169(0.082–0.350)	<0.001					2.026(0.164–25.056)	0.582		
≥2	1	NS	1			NS			1			
Number of total transfusions (>12 vs. ≤12)		NS		NS		NS		NS		NS		NS
Number of RBC transfusion (>7 vs. ≤7)		NS		NS				NS		NS		
Number of PLT transfusion (>7 vs. ≤7)	2.444(1.227–4.868)	0.011	2.279(1.131–4.594)	0.021	2.678(1.266–5.663)	0.010	8.259(3.357–20.318)	<0.001	6.810(2.889–16.053)	<0.001	5.398(2.426–12.011)	<0.001

Abbreviations: HLA, human leukocyte antigen; OR, odds ratio; CI, confidence interval; RBC, red blood cell; PLT, platelet.

### Prevalence of and risk factors associated with the number of antibodies specific for HLA-A, -B, -C, -DR, -DQ and -DP

**Figure 2:**
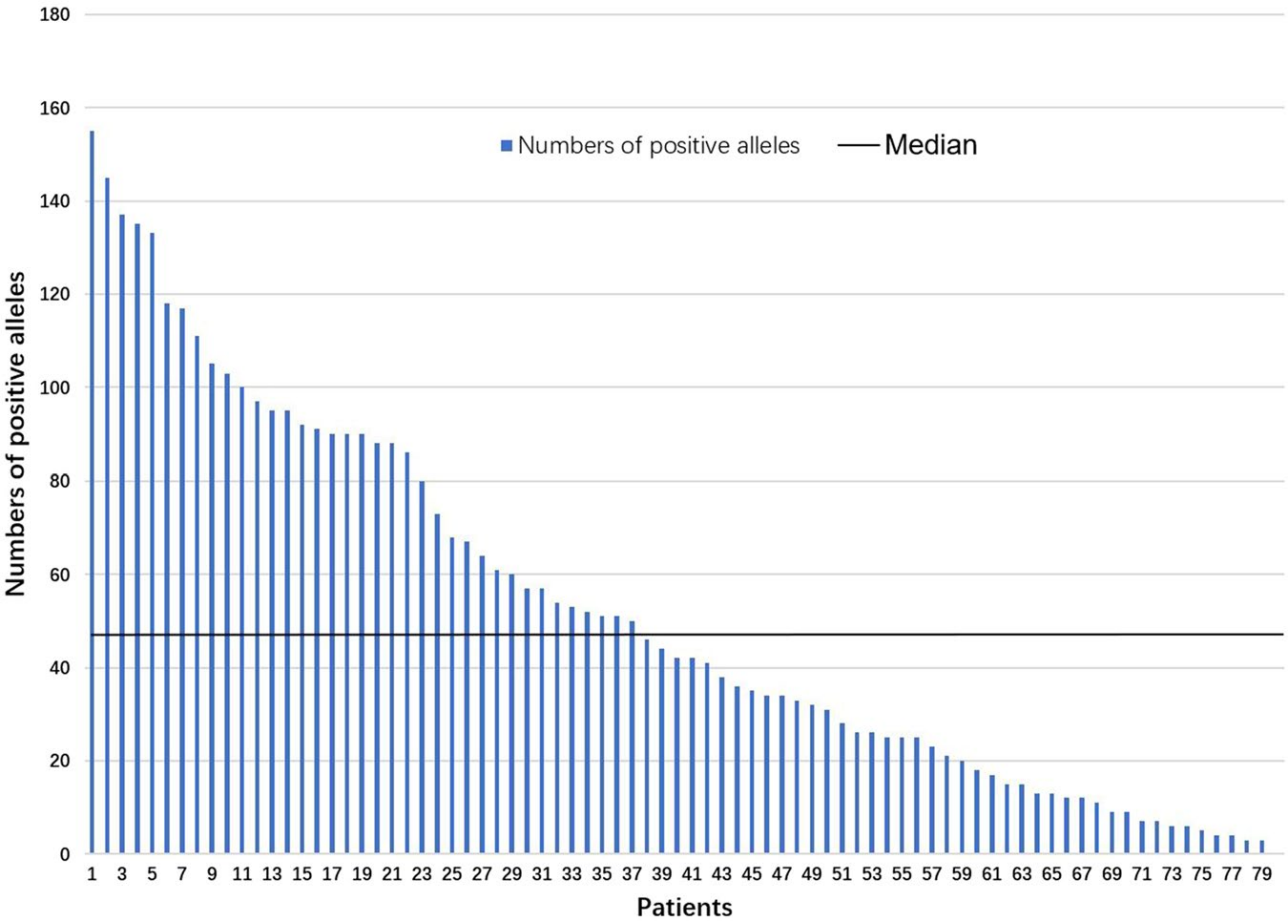
Number of anti-HLA antibodies positive alleles in 79 people.

There were 79 patients with positive anti-HLA antibodies that were specific for single HLA loci, and the median number of antibodies to specific HLA loci was 44, with a range of 3 to 155 (Fig. 2). To investigate the factors associated with the number of anti-HLA antibodies, all 79 patients were classified into two groups according to the median number of antibodies. In univariate analysis, previous total transfusions, RBC transfusions and PLT transfusions were associated with the number of antibodies (Supplementary Table S3). Meanwhile, previous total transfusions (12 or more) (HR 4.041, 95% CI 1.442–11.322, *P* = 0.008) was independently and significantly associated with having higher numbers of anti-HLA antibodies based on multivariate analysis.

### Prevalence of and risk factors associated with MFI of PRAs for class I and class II

There were 73 patients with positive PRAs for class I and 38 patients for class II. A prospective clinical study of our group had concluded that donor-specific anti-HLA antibodies (MFI >/ = 2000) were strongly associated with primary poor graft function (*P* = 0.005)^9^. So we divided patients with MFI of 500 or more into two subgroups according to the cut-off value of 2000. Univariate analysis showed that previous total transfusions, RBC transfusion and disease course were associated with MFI of PRAs for class I, meanwhile, previous total transfusions, PLT transfusions and disease course were associated with MFI of PRAs for class II. Multivariate analysis showed that previous transfusions (12 or more) (HR 3.529, 95% CI 1.119–11.133, *P* = 0.031) and disease course (7.5 months or more) (HR 3.389, 95% CI 1.212–9.477, *P* = 0.020) were related to higher MFI of PRAs for class I. In the meanwhile, PLT transfusions (7 or more) (HR 5.833, 95% CI 1.061–32.020, *P* = 0.042) were correlated with MFI of PRAs for class II in multivariate analysis. (Supplementary Table S4).

## Discussion

In this study, we demonstrated the following: i) female sex was associated with having anti-HLA class II antibodies and antibodies to specific loci, including HLA-C and -DQ; ii) prior pregnancy was a risk factor for having anti-HLA class I antibodies and antibodies to specific loci, including HLA-A, -B and -DQ; and iii) PLT transfusions (7 or more) were related to having anti-HLA class I, and II antibodies as well as antibodies to specific loci, including HLA-A, -B, -C, -DP, -DQ and -DR. In the meanwhile, previous total transfusions were also an independent risk factor for having higher numbers of anti-HLA antibodies and higher MFI of PRAs for class I. In addition, disease course (7.5 months or more) were related to higher MFI of PRAs for class I; while PLT transfusions (7 or more) were related to higher MFI of PRA for class II. The results were consistent with the findings of previous studies^19–21,23^ and are useful for haplo-SCT donor selection.

In this study, the prevalence of anti-HLA antibodies was 28.9% (79/273) among all older patients, which was higher than previous studies of the general population (21%)^21^ and pediatric candidates (13%)^22^ with hematologic disease at our center. De Clippel *et al*.^24^ concluded that in people with a history of transfusion, the overall alloimmunization rate against class I and II HLA antigens was 20.2%. Triulzi *et al*.^25^ found that HLA antibodies were detected in 17.3% of all female volunteer blood donors and 1.7% of transfused male volunteer blood donors. All of the abovementioned proportions of anti-HLA antibodies were lower than those observed in the results of our study. However, Yee *et al*.^26^ found that in pediatric patients with thalassemia major, HLA antibodies were detected in 10 of 19 (53%) subjects. The differences in the proportion of anti-HLA antibodies between our study and others’ studies may be because of the type of disease, sex, age, and the number of previous pregnancies and transfusions.

The association between sex and having anti-HLA antibodies has been previously reported^27–30^. Gladstone *et al*.^30^ retrospectively reviewed 957 donors to evaluate the incidence of anti-HLA antibodies and DSAs and found that the incidence of anti-HLA antibodies was higher among females than males (43.2 versus 10.8%, *P* < 0.0001), and DSAs were most commonly detected in female patients (30.6% versus 4.9%, *P* < 0.00001) among candidates for haplo-SCT. Furthermore, Hyun *et al*.^28^ concluded that female solid organ transplantation candidates had a significantly higher PRA-positivity rate compared with that of male patients (60.3% vs 34.2%; P < 0.001). Nguyen *et al*.^29^ also found that women were at a higher risk of antibody-mediated rejection than men, and this increased risk was additive to that of preformed donor-specific antibodies after heart transplantation. Therefore, sex can be regarded as a specific risk factor for having anti-HLA antibodies and could guide their detection before SCT.

Almost all of the recent studies have confirmed that pregnancy was a risk factor for having anti-HLA antibodies^31^; additionally, our study also showed that pregnancy was not only associated with having PRAs for class I but was also correlated with having antibodies against different antigens encoded by the different HLA loci, including HLA-A, -B and -DQ. Masson *et al*.^32^ showed that allo-immunized mothers presented more anti-HLA class I antibodies more frequently (86%) than they presented anti-class II antibodies (62%). This may explain why pregnancy is a risk factor for PRAs for class I rather than for class II based on the multivariate analysis of our study. In addition, our study showed that the incidences of PRAs for class I and II increased with the number of pregnancies as follows: 7.1% (zero), 14.8% (one) and 25.0% (two or more). Female patients are exposed to antigens during pregnancy^31^, and the exposure intensity increases as the number of pregnancies increases, which suggested to us that postpartum women should be monitored for anti-HLA antibodies routinely, especially among women who have had multiple pregnancies.

In the present study, the association of transfusion with a higher incidence of anti-HLA antibodies was also confirmed. First, PLT transfusions were the risk factors for having PRAs for class I and II, and were correlated with having antibodies against different antigens, including anti-HLA-A, -B, -C, -DP, -DQ, and -DR. Second, when patients had undergone more transfusions, they had higher numbers of antibodies to specific HLA loci. Huo *et al*.^21^ found that prior transfusions (6 or more) is a risk factor associated with the prevalence of having a positive PRA either for class I or class II HLA in haploidentical transplant candidates. Hung *et al*.^19^ observed that having undergone a recent transfusion but not having undergone a transfusion itself was independently correlated with HLA sensitization in patients with end-stage renal disease. However, most studies concluded that transfusion had little impact on anti-HLA antibodies^23–25,33^. In contrast to previous studies^23–25,33^, but in agreement with others^34^, we found that PLT transfusions were not only associated with PRAs for class I, but also correlated to PRAs for class II. The higher white blood cells contained in platelet components than that of the RBC components may account for above mentioned association^35^.

Previous studies have demonstrated that DSAs were associated with GF in both haplo-SCT^9,36,37^ and MUDT^12^, and the incidence of GF increases as the MFI increased^9,36^. In this study, we found that previous total transfusions (12 or more) and disease course (7.5 months or more) were related to higher MFI of PRAs for class I, while PLT transfusions (7 or more) were related to MFI of PRAs for class II. The results in Supplementary Table S4 showed that female, multiple pregnancies, previous total transfusion, RBC transfusions or PLT transfusions and long disease courses had a tendency for high MFI, which need a larger sample size of patients to explore.

This study had several limitations. First, this was a single center study. Second, our research is limited to Chinese populations, and the possibility of extending these findings to other populations, such as white, black, and Hispanic populations, needs to be investigated. Thus, a multicenter study with a large sample size is warranted.

In summary, the results of this study suggested that female sex, prior pregnancy and PLT transfusions are independent risk factors for the generation of anti-HLA antibodies in haploidentical transplant candidates aged more than 50 years. These findings could guide clinical monitoring for HLA antibodies and may contribute to haploidentical donor selection.

## Supplementary information

Supplementary Tables
