# Supplementary material for: Prevalence and risk factors of having antibodies to class I and II human leukocyte antigens in older haploidentical allograft candidates
Source: Sci Rep. 2020 Feb 11;10:2367. doi: 10.1038/s41598-020-59417-1 (PMC7012824; doi:10.1038/s41598-020-59417-1)
Supplement: Supplementary file 1 — Supplementary Tables [file 41598_2020_59417_MOESM1_ESM.pdf]

Prevalence and risk factors of having antibodies to class I and II human leukocyte antigens in older haploidentical allograft candidates

Le-Qing Cao[1], Meng Lv[1], Lan-Ping Xu[1], Xiao-Hui Zhang[1], Huan Chen[1], Yu-Hong Chen[1], Feng-Rong Wang[1], Wei Han[1], Yu-Qian Sun[1], Chen-Hua Yan[1], Fei-Fei Tang[1], Xiao-Dong Mo[1], Kai-Yan Liu[1], Xiao-Jun Huang[1,2,3], and Ying-Jun Chang*[1,3]

[1] Peking University People's Hospital and Peking University Institute of Hematology, Beijing Key Laboratory of Hematopoietic Stem Cell Transplantation, No. 11 South Street of Xizhimen, Xicheng District, Beijing 100044, P.R.C [2]Peking-Tsinghua Center for Life Sciences, Beijing 100871, China, [3] Collabrative Innovation Center of Hematology, Peking University, Beijing 100044, China.

Running title: Risk factors for having anti-HLA antibodies in haplo-SCT

*** Correspondence:**

Ying-Jun Chang, Ph.D
Professor of Peking University Institute of Hematology
Peking University People's hospital
No 11 Xizhimen South Street, Beijing 100044, China
Phone: 86-010-88325949
Fax: 86-010-88324577
E-mail: rmcyj@bjmu.edu.cn

Abstract words: 271 words
Text words: 2615 words

Supplementary materials

Table S1. Univariate analysis for PRA in 273 older allogeneic stem cell transplantation candidates

	Class I (+)		Class II (+)		Class I or II (+)		Class I and II (+)	
	n (%)	*p*	n (%)	*p*	n (%)	*p*	n (%)	*p*
Gender								
Male（n=165）	30(18.2%)	< 0.001	13(7.9%)	< 0.001	32(19.4%)	< 0.001	11(6.7%)	0.001
Female (n=108)	43(39.8%)		25(23.1%)		47(43.5%)		21(19.4%)	
Number of pregnancies								
0 (n=168)	31(18.5%)	< 0.001	14(8.3%)	0.001	33(19.6%)	< 0.001	12(7.1%)	0.006
1 (n=61)	18(29.5%)		10(16.4%)		19(31.1%)		9(14.8%)	
⩾2 (n=44)	24(54.5%)		14(31.8%)		27(61.4%)		11(25.0%)	
Number of total transfusions								
≤12(n=212)	50(23.6%)	0.028	20(9.4%)	< 0.001	54(25.5%)	0.019	16(7.5%)	< 0.001
>12 (n=61)	23(37.7%)		18(29.5%)		25(41.0%)		16(26.2%)	
Number of RBC transfusion								
≤7(n=212)	51(24.1%)	0.062	20(9.4%)	0.071	55(25.9%)	0.042	16(7.5%)	< 0.001
>7 (n=61)	22(36.1%)		18(29.5%)		24(39.3%)		16(26.2%)	
Number of PLT transfusion								
≤7(n=226)	53(23.5%)	0.007	22(9.7%)	< 0.001	57(25.2%)	0.003	18(8.0%)	< 0.001
>7 (n=47)	20(27.4%)		16(34.0%)		22(46.8%)		14(29.8%)	
Course								
≤7.5 (n=169)	39(23.1%)	0.081	20(11.8%)	0.205	42(24.9%)	0.058	17(10.1%)	0.276
>7.5(n=104)	34(32.7%)		18(17.3%)		37(35.6%)		15(14.4%)	

Abbreviations: PRA, panel reactive antibody; RBC, red blood cell; PLT, platelet.

Table S2. Specific antibodies against HLA-A, -B, -C, -DP, -DQ, and -DR: Univariate analysis in 273 older allogeneic stem cell transplantation candidates

	anti-A (+)		anti-B (+)		anti-C (+)		anti-DP (+)		anti-DQ (+)		anti-DR (+)	
	n (%)	*p*	n (%)	*p*	n (%)	*p*	n (%)	*p*	n (%)	*p*	n (%)	*p*
Gender												
Male（n=165）	29(17.6%)	0.001	27(16.4%)	< 0.001	18(10.9%)	0.002	10(6.1%)	0.082	11(6.7%)	0.003	13(7.9%)	0.001
Female (n=108)	38(35.2%)		43(39.8%)		27(25.0%)		13(12.0%)		20(18.5%)		24(22.2%)	
Number of pregnancies												
0 (n=168)	30(17.9%)	< 0.001	28(16.7%)	< 0.001	19(11.3%)	0.005	11(6.5%)	0.051	12(7.1%)	0.001	14(8.3%)	0.001
1 (n=61)	16(26.2%)		18(29.5%)		12(19.7%)		4(6.6%)		6(9.8%)		9(14.8%)	
⩾2 (n=44)	21(47.7%)		24(54.5%)		14(31.8%)		8(18.2%)		13(29.5%)		14(31.8%)	
Number of total transfusions												
⩽12 (n=212)	45(21.2%)	0.018	47(22.2%)	0.014	31(14.6%)	0.122	9(4.2%)	< 0.001	15(7.1%)	< 0.001	19(9.0%)	< 0.001
>12 (n=61)	22(36.1%)		23(37.7%)		14(23.0%)		14(23.0%)		16(26.2%)		18(29.5%)	
Number of RBC transfusion												
⩽7 (n=212)	46(21.7%)	0.042	48(22.6%)	0.034	33(15.6%)	0.446	10(4.7%)	< 0.001	16(7.5%)	< 0.001	19(9.0%)	< 0.001
>7 (n=61)	21(34.4%)		22(36.1%)		12(19.7%)		13(21.3%)		15(24.6%)		18(29.5%)	
Number of PLT transfusion												
⩽7 (n=226)	48(21.2%)	0.005	51(22.6%)	0.011	31(13.7%)	0.007	10(4.4%)	< 0.001	16(7.1%)	< 0.001	21(9.3%)	< 0.001
>7 (n=47)	19(40.4%)		19(40.4%)		14(29.8%)		13(27.7%)		15(31.9%)		16(34.0%)	
Course												
⩽7.5 (n=169)	37(21.9%)	0.195	37(21.9%)	0.071	24(14.2%)	0.195	10(5.9%)	0.057	15(8.9%)	0.100	19(11.2%)	0.155
>7.5(n=104)	30(28.8%)		33(31.7%)		21(20.2%)		13(12.5%)		16(15.4%)		18(17.3%)	

Abbreviations: HLA, human leukocyte antigen; RBC, red blood cell; PLT, platelet.

Table S3. Univariate analysis in 79 anti-HLA antibody positive older patients

	≤44 HLA loci	>44 HLA loci	
	n (%)	n (%)	*p*
Gender			
Male（n=31）	13(41.9%)	18(58.1%)	0.247
Female (n=47)	26(55.3%)	21(44.7%)	
Number of pregnancies			
0 (n=33)	14(42.4%)	19(57.6%)	
1 (n=19)	13(68.4%)	6(31.6%)	0.186
⩾2 (n=27)	13(48.1%)	14(51.9%)	
Number of transfusions			
≤12 (n=54)	33(61.1%)	21(38.9%)	0.006
>12(n=25)	7(28.0%)	18(72.0%)	
Number of RBC transfusion			
≤7(n=212)	32(58.2%)	23(41.8%)	0.042
>7 (n=61)	8(33.3%)	16(66.7%)	
Number of PLT transfusion			
≤7(n=226)	34(59.6%)	23(40.4%)	0.010
>7 (n=47)	6(27.3%)	16(72.7%)	
Course			
≤7.5 (n=42)	22(52.4%)	20(47.6%)	0.741
>7.5 (n=28)	18(48.6%)	19(51.4%)	

Abbreviations: HLA, human leukocyte antigen; RBC, red blood cell; PLT, platelet.

Table S4. Univariate analysis in 73 patients having a positive PRA for class I and 38 patients having a positive PRA for class II with MFI⩾500.

	Class I (n %)			Class II (n %)		
	500⩽MFI < 2000	MFI⩾2000	*P*	500⩽MFI < 2000	MFI⩾2000	*P*
Gender			0.280			0.525
Male	15 (50.0%)	15 (50.0%)		3 (23.1%)	10 (76.9%)	
Female	16 (37.2%)	27 (62.8%)		9 (36.0%)	16 (64.0%)	
Number of pregnancies			0.181			0.466
0	15 (48.4%)	16 (51.6%)		3 (21.4%)	11 (78.6%)	
1	9 (50.0%)	9 (50.0%)		4 (40.0%)	6 (60.0%)	
⩾2	7 (29.2%)	17 (70.8%)		5 (35.7%)	9 (64.3%)	
Number of transfusions			0.057			0.064
≤12	25 (50.0%)	25 (50.0%)		9 (45.0%)	11 (55.0%)	
>12	6 (26.1%)	17 (73.9%)		3 (16.7%)	15 (83.3%)	
Number of RBC transfusion			0.085			0.239
≤7(n=212)	25(49.0%)	26(51.0%)		8(40.0%)	12(60.0%)	
>7 (n=61)	6(27.3%)	16(72.7%)		4(22.2%)	14(77.8%)	
Number of PLT transfusion			0.186			0.031
≤7(n=226)	25(47.2%)	28(52.8%)		10(45.5%)	12(54.5%)	
>7 (n=47)	6(30.0%)	14(70.0%)		2(12.5%)	14(87.5%)	
Course			0.036			0.064
≤7.5	21 (53.8%)	18 (46.2%)		9 (45.0%)	11 (55.0%)	
>7.5	10 (29.4%)	24 (70.6)		3 (16.7%)	15 (83.3%)	

Abbreviations: HLA, human leukocyte antigen, PRA, panel-reactive antibody, MFI, mean fluorescence intensity; RBC, red blood cell; PLT, platelet.
